# Association of Patient, Prescriber, and Region With the Initiation of First Prescription of Biologic Disease-Modifying Antirheumatic Drug Among Older Patients With Rheumatoid Arthritis and Identical Health Insurance Coverage

**DOI:** 10.1001/jamanetworkopen.2019.17053

**Published:** 2019-12-06

**Authors:** Mark Tatangelo, George Tomlinson, J. Michael Paterson, Vandana Ahluwalia, Alex Kopp, Tara Gomes, Nick Bansback, Claire Bombardier

**Affiliations:** 1 University of Toronto, Toronto, Ontario, Canada; 2 University Health Network, Toronto, Ontario, Canada; 3 ICES, Toronto, Ontario, Canada; 4 William Osler Health Care Centre, Brampton, Ontario, Canada; 5 St Michael’s Hospital, Toronto, Ontario, Canada; 6 University of British Columbia, Vancouver, British Columbia, Canada

## Abstract

**Importance:**

Prescribing the first biologic treatment for rheumatoid arthritis (RA) is an important decision for patients, their physicians, and payers, with considerable costs and clinical implications. Conventional synthetic disease-modifying antirheumatic drugs (csDMARDs) have known effectiveness and safety profiles and are less expensive; therefore, determining the variables contributing to csDMARD treatment duration is an essential question for patients, physicians, and payers.

**Objectives:**

To describe access to the first biologic DMARD prescription in a population of patients with RA and identical comprehensive health insurance coverage in Ontario, Canada, and to explore the associations of patient, prescriber, and geographic region with differences in time to first biologic prescription.

**Design, Setting, and Participants:**

This cohort study of incident patients with RA used administrative data with surveillance and patient-level data collected at yearly intervals. A total of 17 672 patients were included in the study; they were residents of Ontario, Canada, had an incident RA diagnosis at age 67 or older between 2002 and 2015, and received at least 1 csDMARD. Data were analyzed in November 2017.

**Exposure:**

Patient variables were age, sex, disease duration, socioeconomic status, distance to care, and supply of care in the patient’s area of residence. Prescriber covariates were year of graduation, specialty of practice, and supply of rheumatologic care in the patient’s geographic region.

**Main Outcomes and Measures:**

Time from first csDMARD prescription to receipt of first biologic medication.

**Results:**

Of 17 672 patients, 11 598 (65.6%) were women, and the mean (SD) age was 75.2 (5.8) years. Characteristics associated with longer time to receipt of a biologic prescription were older age (HR for every 5-year increase, 0.66; 95% CI, 0.62-0.71; *P* < .001), male sex (HR, 0.76; 95% CI, 0.66-0.89; *P* < .001), and distance to the nearest rheumatologist (HR per 10-km increase, 0.99; 95% CI, 0.98-0.99; *P* < .001). Prescribers were primarily rheumatologists (151 of 214 [70.6%]) and primary care physicians (26 of 214 [12.1%]). After adjusting for the number of patients eligible to receive biologic DMARDs, rheumatologists’ preferences (ie, yearly prescription rates) for using biologic DMARDs increased over time, from 1.7% in 2001 to 4.9% in 2015. After adjusting for calendar year and patient-, prescriber-, and region-level characteristics, substantial variation between prescribers in rates of prescribing a first biologic DMARD were found (65% variance).

**Conclusions and Relevance:**

This study found variation in time to receipt of first biologic DMARD after prescription of first csDMARD in a population with RA after adjustment for individual-level patient, prescriber, and geographic area covariates, despite identical universal health insurance coverage.

## Key Points

**Question:**

What patient, prescriber, and regional factors are associated with time to first prescription of biologic disease-modifying antirheumatic drug (DMARD) among patients 67 years or older with rheumatoid arthritis?

**Findings:**

In this cohort study of 17 672 older patients with rheumatoid arthritis, patients were more likely to receive biologic DMARDs earlier if they were younger, female, and living in urban areas closer to prescribers. Physician preference was strongly associated with differences in time from first conventional synthetic DMARD to first biologic DMARD.

**Meaning:**

In this study, pharmacologic care for rheumatoid arthritis was not uniform across patients and prescribers given similar disease characteristics in a population with identical health insurance coverage.

## Teaser

This cohort study evaluates the associations of patient, prescriber, and geographic region with differences in time to first prescription of biologic disease-modifying antirheumatic drug among older adults with rheumatoid arthritis and identical health insurance coverage in Ontario, Canada.

## Introduction

In Canada, approximately 300 000 patients, or 1% of the population, are living with rheumatoid arthritis (RA).^1^ Treatment advances during the past 20 years have radically improved participation and quality-of-life outcomes for patients with access to evidence-informed care. These advances have included high-quality evidence to support the use of conventional synthetic disease-modifying antirheumatic drugs (csDMARDs) at optimal dosing combinations and thresholds, and the development of biologic DMARDs, which specifically target the inflammatory mechanisms of RA.

Switching to biologic therapy for RA is an expensive health care decision for patients, physicians, and payers. Optimizing dose and treatment time with first-line csDMARDs saves health care resources, with a difference in costs of more than CaD$15 000 (US$11 460) annually, while preserving the clinical choice to escalate treatment to biologic therapy.^2,3,4^ The ability to escalate to a biologic DMARD is critical; all clinical practice guidelines for RA recommend an adequate trial of csDMARDs before initiating a biologic medication.^2,3,4^ Despite these recommendations, limited evidence informs clinicians and policy makers on the factors associated with earlier receipt of a biologic DMARD. This limitation is troubling because wide variations in access to and time to initiation of biologic therapy have been observed across health care jurisdictions.^5,6,7,8,9,10,11,12,13,14,15,16,17,18,19,20^

There are several challenges to research in this area. Research on the initiation of the first biologic medication is often limited by small nonrepresentative samples and short follow-up periods, reducing the interpretability and generalizability of findings.^21,22^ In addition, prescriber decisions to switch to biologic DMARDs are often influenced by nonmedical reasons associated with a patient’s insurance coverage.^8,9^ Variations in insurance coverage within patient populations confound clinical studies and limits causal attribution. Furthermore, patient, prescriber, and regional characteristics are often analyzed individually instead of simultaneously, a method that does not reflect the complexity of treatment decision-making in usual care.^23,24^ The objectives of this study were to describe access to the first biologic DMARD prescribed in a population of patients with RA and identical comprehensive health insurance coverage in Ontario, Canada, and to explore the associations of patient, prescriber, and geographic region with differences in time to first prescription of biologic DMARD.

## Methods

### Study Design and Setting

We conducted a retrospective cohort study using deidentified population-based administrative health care databases from ICES (formerly the Institute for Clinical Evaluative Sciences).^25^ All residents of Ontario aged 65 years and older are covered under the Ontario Health Insurance Plan, which includes physician visits, acute care hospital use, and all prescriptions. Research ethics committee approval was received from the University Health Network, Toronto, Canada, with individual consent waived in accordance with local privacy laws granted by section 45 of Ontario’s Personal Health Information Privacy Act. This report follows the Strengthening the Reporting of Observational Studies in Epidemiology (STROBE) reporting guideline for cohort studies.^26^

### Data Sources

Data sources included the Registered Persons Database for patient demographic data, Ontario Health Insurance Plan claims for physician visits, the Discharge Abstract Database for inpatient hospitalization records, and the Ontario Drug Benefit claims database for prescription medication dispensation. Prescriber variables were obtained from the ICES Physician Database. The Immigration Refugees and Citizenship Canada Database contained immigration status information for our study.

### Inclusion Criteria

Patients were included if they were a resident of Ontario with a valid health card number and an RA diagnosis, which was identified using a validated algorithm. The incident date of RA diagnosis was determined as the date on which the final billing criteria was met (ie, 3 RA billings within 2 years, with ≥1 billing by a musculoskeletal specialist or ≥1 hospitalization for RA).^1^ To be eligible for analytic models, patients had to have incident onset RA at age 67 or older and be csDMARD naive. For unadjusted descriptive analysis, active users older than 65 years were included from 2001 to 2015, and unadjusted survival models used patients accrued from 2002 to 2015, with a maximum follow-up to 2017.

### Washout Period

This population had a 2-year study washout period (ie, covering ages 65 and 66 years) to ensure the capture of all newly administered csDMARDs and biologic DMARDs since diagnosis of RA in the biologics era (ie, after 2001). An additional year of washout from 2001 to 2002 was used to remove potential bias from patients who may have immediately switched to biologic DMARDs when they received approval from regulators.

### Time to First Biologic DMARD

To be eligible for the analysis of time from first csDMARD to first biologic DMARD, patients had to have received at least 1 csDMARD or biologic DMARD with an approved indication for RA. A full list of medications used in this analysis is provided in the eAppendix in the Supplement. The small-molecule selective inhibitor tofacitinib was excluded because fewer than 10 patients had an exposure during the observation window.

### Observation Window

The study observation window was 2002 to 2015, with accrual ending in 2014, ensuring at least 1 complete year of follow-up for each patient (eFigure 1 in the Supplement). Data were analyzed in in November 2017, allowing a 2-year window to ensure all data were captured from each data source.

### Patient Characteristics

Patient demographic variables were age, sex, disease duration, geographic area of residence, distance to prescriber of each medication, and distance to nearest rheumatologist. Health care resource use was operationalized using the Johns Hopkins Adjusted Comorbidity Group version 10,^27^ rurality index (range, 0-100; 0 indicates most urban; 100, most rural),^28^ neighborhood income quintile,^29^ and marginalization index.^30^ Neighborhood income quintile was calculated using national census data to set income quintile distribution with geographic assignment based on dissemination areas containing 500 to 800 people each. The marginalization index was also based on the characteristics of dissemination areas, focusing on 4 dimensions that contribute to the process of marginalization: residential instability, material deprivation, dependency, and ethnic concentration. Age and sex were considered fixed variables, with disease duration considered a time-varying (count) variable and calendar year considered a categorical variable. All patient variables except for age, sex, and disease duration were time-varying by calendar year.

### Prescriber Characteristics

For each patient, prescriber variables were assigned by unique physician identifier number at the first new csDMARD or biologic DMARD prescription dispensation and carried forward to all subsequent prescriptions of the same medication. Therefore, the characteristics of the first prescriber of each new RA medication were assigned for each patient in the time-varying model. Biologic DMARD prescribers (eTable in the Supplement) were operationalized as physicians who had prescribed at least 10 biologic treatments during the study period. Prescriber characteristics were age, practice location, practice size, year of graduation, and specialty. Preference for biologic DMARD prescription for each physician was computed by dividing the number of times the clinician switched a biologic-naive patient to a biologic DMARD each year by the total number of biologic-naive patients in each year. This variable adjusts for physician-level preference for prescribing a biologic DMARD in a given year, while reflecting changing physician preferences over time.

### Regional Characteristics

Access to physicians was estimated by computing the linear distance from patient to prescriber of each medication and from patient to the nearest available rheumatologist. To concurrently adjust for rheumatologist care in relation to regional demand for rheumatologists, we calculated the quantity-adjusted supply of rheumatologists,^31^ a new rheumatologist supply variable. This was calculated by dividing the annual number of rheumatologist visits in each region by the annual number of patients with RA who resided in each region. Rurality and socioeconomic status of physician practices were measured using rurality score^28^ and neighborhood income quintile.^29^ We operationalized 14 geographic regions using the Local Health Integration Network, an administrative geographic stratifier for health care delivery in Ontario^32^ (eFigure 2 in the Supplement).

### Outcome, Exposure, and Covariate Definitions

The primary outcome for the explanatory analytic models was an intent-to-treat analysis from the time from the first csDMARD prescription to receipt of a biologic DMARD approved for RA, with censoring at death, loss of eligibility for public health care (ie, moved out of Ontario), or the end of follow-up (ie, December 31, 2015). Model explanatory exposure variables, including patient-level, physician-level, and region-level factors, were measured at yearly intervals, with medications recorded at each pharmacy dispensation and physician demographic characteristics updated every 90 days.

### Missing Data

There were no missing patient characteristic variables or medication prescription variables; however, 18 571 of 385 612 physician characteristic variables (4.8%) attached to prescription data were missing because of pharmacy omission. Observations with a missing prescriber identification number value were assigned the identification number of the identical prescription closest in time for the patient.

### Statistical Analysis

To illustrate the unadjusted association of region with receipt of first biologic DMARD, Kaplan-Meier curves stratified by region were calculated. Time from first csDMARD prescription to first biologic DMARD prescription was the outcome in mixed-effects Cox proportional hazard models, with random effects for region and physician nested within regions^33^ (eTable in the Supplement). This nested model choice reflects usual care; patients are prescribed medications by physicians who practice within regions. Time-dependent covariates were measured based on the covariates of the first prescriber of the csDMARD or biologic DMARD and the patient’s characteristics at time of prescription. Models also accommodated crossed random effects; patients could switch physicians, and physicians could move practice locations. Therefore, although models were nested, models were flexible to account for patients changing physicians or moving between regions, which reflects the realities of usual clinical care. To adjust for changing numbers of available biologic medications over time and year-specific factors, a categorical variable for calendar year was added to the model.

Outcomes were measured at the patient level and reported as hazard ratios (HRs) with 95% CIs and *P* values. We used 2-tailed *P* < .05 as the threshold for statistical significance. From first csDMARD prescription, patient’s prescriptions were divided into a sequence of intervals based on the drug prescribed to accommodate time-varying patient, prescriber, and regional covariates. At the end of the follow-up period, each patient was either censored or had received a biologic DMARD, meaning trends in physician preference for biologic use were explicitly measured. A simple model showing the unadjusted association of the exposure with the outcome was contrasted with a full model, adjusting for all variables in the simple model as well as random effects, to calculate the association of physician variation with the time to receipt of first biologic DMARD. All analysis was performed using R statistical software version 3.1.2 (R Project for Statistical Computing).^34^

## Results

A total of 17 672 patients met the study inclusion criteria, accruing 82 445 patient-years of follow-up (Table).^35^ A total of 719 patients (4.1%) received a first biologic prescription during the study.

**Table.** Effect Estimates From Patient and Prescriber Variables

Characteristic	Baseline Covariates^a^		Fully Adjusted Cox Model		
	Mean (SD)	Median (IQR) [Range]	HR (95% CI)	SE	*P* Value
**Characteristics for 17 672 Patients**					
Men, No. (%)	6074 (34.4)	NA	0.76 (0.66-0.89)	0.08	<.001
Age, per 5-y increase, y	75.2 (5.8)	74 (70-79) [67-99]	0.66 (0.62-0.71)	0.01	<.001
Linear distance to prescriber, per 10-km increase, km	125.50 (304.37)	11.60 (4.37-53.62) [0-3016]	1.01 (1.00-1.02)	0.01	<.001
Linear distance to nearest rheumatologist, per 10-km increase, km	129.40 (309.15)	11.99 (4.44-56.39) [0-3016]	0.99 (0.98-0.99)	0.01	<.001
Calendar year, per 5-y increase	2009 (3.91)	2009 (2006-2013) [2002-2015]	0.49 (0.43-0.55)	0.01	<.001
Rurality index, per 20-unit increase	11.86 (18.51)	2 (0-20) [0-100.00]	1.09 (0.99-1.05)	0.05	.06
Disease duration, per 5-y increase	NA	NA	1.35 (1.19-1.54)	0.01	<.001
Marginalization index, per unit increase	3.12 (0.78)	3.00 (2.50-3.75) [1-5]	0.99 (0.87-1.11)	0.06	.81
Neighborhood income quintiles	3.06 (1.39)	3.00 (2.00-4.00) [1.00-5.00]	0.92 (0.65-1.31)	0.03	.16
1	NA	NA	0.92 (0.65-1.31)	0.03	.16
2	NA	NA	1.07 (0.83-1.37)	0.28	.14
3	NA	NA	1.19 (0.91-1.55)	0.33	.16
4	NA	NA	1.07 (0.81-1.42)	0.31	.16
5	NA	NA	1.27 (0.95-1.71)	0.39	.19
Immigrant, No. (%)^b^	1131 (6.40)	NA	0.59 (0.42-0.84)	0.18	.003
Hopkins Adjusted Comorbidity Group score^c^	4749 (435.30)	4910 (4420-4930) [300-5200]	1.00 (0.99-1.01)	0.01	.48
**Characteristics of First Prescriber of csDMARD**					
Graduation year, per 5-y increase	1984 (11.33)	1984 (1977-1994) [1942-2013]	1.10 (1.04-1.17)	0.01	.001
Women, No. (%)^d^	5703 (32.3)	NA	0.97 (0.74-1.27)	0.14	.83
Rurality index score, per 20-unit increase	4.80 (11.22)	0 (0-5) [0-100]	0.67 (0.50-0.92)	0.01	.01
Rheumatologist, No. (%)	12 771 (72.2)	NA	0.92 (0.65-1.31)	0.01	.66
Quantity-adjusted supply of rheumatologists	4.14 (2.72)	3.78 (2.36-5.13) [0-16.69]	1.03 (1.00-1.06)	0.02	.03

Abbreviations: HR, hazard ratio; IQR, interquartile range; NA, not applicable.

^a^ Incident rheumatoid arthritis diagnoses among patients 67 years and older after 2001.

^b^ Available since 1985, these data underestimate immigration status by approximately 30%.^35^

^c^ Higher score indicates more comorbidities.

^d^ Data on sex were missing for 536 prescribers, so sex was imputed using the prescriber sex percentage.

### Differences in Biologic DMARD Prescription by Patient Characteristics

#### Patient Demographic Characteristics

Patients who received a csDMARD after RA diagnosis at 65 years or older had a mean (SD) age of 75.2 (5.8) years at baseline, were predominantly women (11 598 women [65.6%]), and tended to reside in more urban areas (mean [SD] rurality score, 11.86 [18.51]). Immigrants represented 1131 patients (6.4%) receiving csDMARDs at baseline (Table).

**Figure 1.**
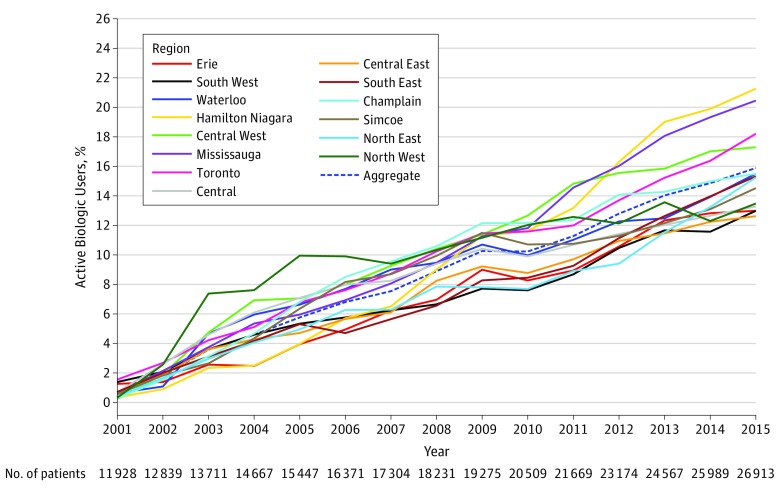
Active Biologic Prescription Percentage as a Proportion of Active Users of All Disease-Modifying Antirheumatic Drugs (DMARDs) Among Older Adults With Rheumatoid Arthritis Unadjusted proportion of all patients older than 65 receiving biologic DMARD for rheumatoid arthritis, by region.

**Figure 2.**
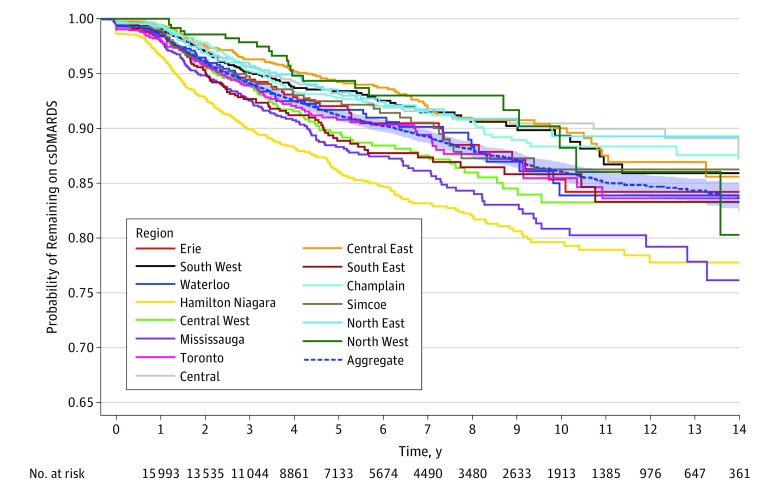
Time From First Conventional Synthetic Disease-Modifying Antirheumatic Drug (csDMARD) to First Biologic DMARD Among Older Adults With Rheumatoid Arthritis in Ontario, Canada, Stratified by Region Unadjusted survival curves for patients older than 67 years who started their first csDMARD prescription between 2002 and 2015, inclusive, with a maximum follow-up to 2017. The shaded area around the aggregate curve represents the 95% CI.

In unadjusted descriptive analysis of all active medication users, disparities in biologic prescription percentage between regions increased over time in Ontario (Figure 1). In 2002, the difference between the highest and the lowest regional percentages of biologic DMARDs used per person was 1.8 percentage points (Toronto, 2.7% vs Central East, 0.9%; difference, 68.4%). In 2015, the difference between the highest and the lowest regional percentages was 8.7 percentage points (Hamilton Niagara, 21.3% vs Central East, 12.6%; difference, 66.3%). Unadjusted Kaplan-Meier curves stratified by region and as an aggregate of all regions illustrated significant differences in time to first biologic DMARD depending on region of patient residence (log-rank test, χ^2^_13_ = 147; *P* < .001) (Figure 2).

Calendar year was associated with time to first biologic DMARD in the fully adjusted Cox model (HR per 5-year increase in calendar year, 0.49; CI, 0.43-0.55; *P* < .001) (Table). For every 5-year increase in age at csDMARD initiation, patients were less likely to receive a biologic DMARD (HR, 0.66; 95% CI, 0.62-0.71; *P* < .001). Men (HR, 0.76; 95% CI, 0.66-0.89; *P* < .001) were also less likely than women to receive biologic medications. Patients who had a longer disease duration were more likely to receive a biologic DMARD (HR per 5-year increase in disease duration, 1.35; 95% CI, 1.19-1.54; *P* < .001). Health care resource use, represented by a higher Hopkins Adjusted Comorbidity Group score, had no observable association with time to receipt of first biologic DMARD (HR, 1.00; 95% CI, 0.99-1.01; *P* = .48) (Table).

#### Socioeconomic Factors

After measuring patient-level variables, including neighborhood income quintile, marginalization index composite scores, immigrant status, and supply of care variables (ie, distance to prescriber), we found mixed results for the association of socioeconomic factors with time to receipt of first biologic DMARD. Living in a neighborhood that belonged in income quintile 1 (HR, 0.92; 95% CI, 0.65-1.31; *P* = .16) and marginalization index composite score (HR, 0.99; 95% CI, 0.87-1.11; *P* = .81) were not statistically significant factors associated with receipt of first biologic prescription. Immigrants were statistically significantly less likely to receive biologic prescriptions (HR, 0.59; 95% CI, 0.42-0.84; *P* = .003) (Table). Greater distance to medication prescriber, irrespective of specialty, was associated with slight increase in likelihood of receiving a biologic prescription (HR per 10-km increase, 1.01; 95% CI, 1.00-1.02; *P* < .001), while patients with a longer distance to the nearest rheumatologist, who may not be the rheumatologist the patient was seeing, were less likely to receive a biologic DMARD (HR per 10-km increase, 0.99; 95% CI, 0.98-0.99; *P* < .001) (Table).

### Differences in Biologic DMARD Prescription by Prescriber Characteristics

#### Prescriber Demographic Characteristics

**Figure 3.**
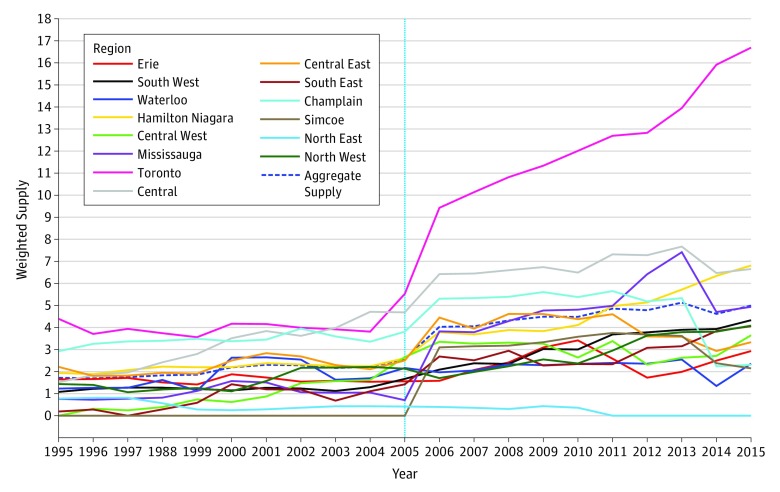
Quantity-Adjusted Supply of Rheumatologists by Region Vertical line represents the change in rheumatologist billing identifier code.

A total of 214 unique prescribers had written 10 or more biologic prescriptions during the study period. Prescribers were primarily rheumatologists (151 of 214 [70.6%]) and primary care physicians (26 of 214 [12.1%]). Among 405 321 prescriptions of biologic DMARDs and csDMARDs initiated or maintained during the study, 291 162 (71.8%) were by a rheumatologist, 56 470 (13.9%) by a primary care physician, 38 306 (9.40%) by an internist, and 16 461 (4.0%) by other specialists. Prescriptions of biologic DMARDs were predominantly initiated or maintained by rheumatologists (1629 of 1977 prescriptions [82.4%]), but primary care physicians (164 prescriptions [8.3%]), internal medicine physicians (55 prescriptions [2.8%]), and dermatologists (23 prescriptions [1.3%]) also prescribed biologic DMARDs in amounts greater than 1% of all biologic prescriptions. Having more recently graduated from medical school was associated with higher propensity to prescribe first biologic DMARD (HR per 5-year after graduation, 1.10; 95% CI, 1.04-1.17; *P* < .001), while women were not statistically different from men in propensity to initiate biologic treatment (HR, 0.97; 95% CI, 0.74-1.27; *P* = .83). Physicians practicing in more rural areas were negatively associated with prescription of biologic DMARDs (HR per 20-unit increase in rurality score, 0.67; 95% CI, 0.50-0.92; *P* = .01), while a greater quantity-adjusted supply of rheumatologists score was associated with a higher likelihood of receipt of biologic DMARDs (HR, 1.03; 95% CI, 1.00-1.06; *P* = .03) (Table and Figure 3).

#### Rheumatologist Preference and Regional Variation

**Figure 4.**
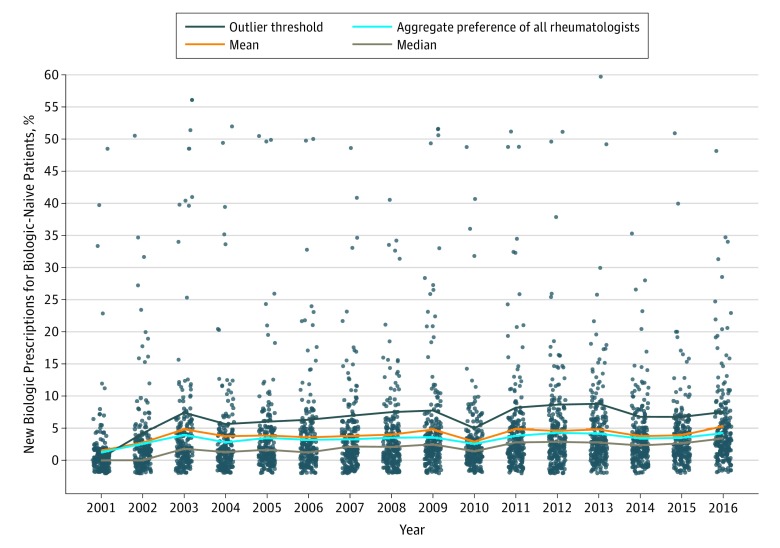
Biologic Prescription Preferences Among 270 Physicians Who Prescribed Biologic Disease-Modifying Antirheumatic Drugs Over Time Preference computed using all available patients. Dots represent individual physicians.

Rheumatologist preferences for prescribing the first biologic DMARD among biologic-naive patients increased by a factor of 3 from 2001 through 2015, from 1.7% to 4.9%, adjusted for the number of patients eligible for receipt of first biologic DMARD (Figure 4). In models adjusted for age, sex, and calendar year, prescriber variance accounted for 74% of the variance in time from first csDMARD to first biologic DMARD (eTable in the Supplement). After adjustment for all patient and physician covariates, physician preference accounted for 65% of the between-region differences, while differences between the regions themselves contributed 4.6% to the overall prescription variation. The remaining 30.4% was owing to underlying variance (eTable in the Supplement).

## Discussion

In a system in which all patients received identical comprehensive prescription coverage, differences in time to receipt of first biologic DMARD persisted after adjustment for individual patient, prescriber, and regional characteristics. Modest between-region variations for time to first biologic prescription were discovered concurrently with comparatively large between-prescriber variations. A 2015 study^36^ showed that several factors, including a complex disease course, multiple health insurers, patient preferences, and the prescribing behaviors of physicians, were associated with differences in time to prescription of the first biologic DMARD, highlighting the importance of nested analysis. The results of this study are similar to the biologic penetration rate of Sweden, where approximately 4% of older patients receive biologic DMARDs.^37^ In our study, the biologic penetration rate of 4.1% (719 of 17 692) was almost identical to the results from Sweden. Our nested adjustment for patients, prescribers, and region provided a complete evaluation of the relative association of a multitude of factors with time to first biologic prescription and demonstrated persistent variations between geographic regions. A 4.6% difference in time to receipt of biologic DMARDs between regions should be considered problematic in the absence of other explanatory factors. For example, every 1% increase in biologic prescriptions among a population of 72 000 funded patients with RA costs approximately CaD$10.8 million (US$8.25 million) per year (assuming 10% biologics penetration and CaD$15 000 annually per biologic DMARD prescription [US$11 460]).^38^ To bring the highest spending region in Ontario in line with the average spend would save approximately CaD$6 million to $8 million (US$4.6 million to $6.1 million) per year, accounting for 16% of the total RA biologic budget and 0.32% of the total all-cause drug formulary budget. Conversely, it would cost approximately CaD$6 million to $8 million (US$4.6 million to $6.1 million) to increase biologic penetration in the underserviced regions of Ontario to meet the population average of all regions.

A higher quantity-adjusted supply of rheumatologists and a more urban rheumatology practice were associated with the receipt of biologic DMARDs. Patients residing in areas with lower socioeconomic status and in rural areas with less access to rheumatologists as well as patients who were immigrants were less likely to receive biologic prescriptions, suggesting a sociodemographic gradient of care for patients with more disadvantage. This study indicated that patients with lower socioeconomic status were the least likely to receive biologic DMARDs. This finding is surprising considering that other published studies show that socioeconomic status is a known risk factor for more severe disease.^39,40,41,42^ The findings of this study suggest that having local access to a rheumatologist may help extend the effective treatment course of csDMARDs. Our findings also suggest that patients may be more willing to travel longer distances to seek biologic treatments. The association between increasing age and greater time to receipt of a biologic DMARD suggested a continuing bias against providing biologic DMARDs for older patients, despite few safety signals indicating a higher risk of infection or other serious adverse events and infections.^37,43^

### Strengths and Limitations

Strengths of this study were the combination of high-quality data and statistical methods. The administrative databases used for exposure, outcome, and covariate definition were validated across diseases and contained uniform data capture and follow-up. The statistical models used were innovative in assigning time-varying exposure to the prescribing physician instead of relying on proxy exposures, in which physician covariates are fixed based on the first prescriber of a csDMARD. In addition, the inclusion of nested random effects for clinicians within regions allowed us to estimate the variation associated with region and clinician. Furthermore, a new variable, the quantity-adjusted supply of rheumatologists, was created to reflect the available supply of rheumatologists in relation to the demand for their care. This variable has a strong theoretical basis in existing economic literature with potential applications to other studies of disparities in health care service delivery.^23,24^

This study also had limitations, including restricting the analysis to patients 67 years or older, an age group in which the propensity to prescribe biologic DMARDs is known to be lower. Owing to higher biologic prescription percentage (ie, 30% estimate^37^) in the population of younger patients with RA, combined with lack of universal publicly funded insurance for patients younger than 65 years in Ontario, we expect that the resulting disparities in biologic prescriptions could be more severe among patients younger than 65 years. The study contained a large sample of patients with RA who live in Canada because Ontario is home to 38% of Canada’s population.^35^ With half of patients with RA being older than 65 years, approximately 19% of all patients with RA in Canada were included in the study. Inherent limitations of administrative data were also present. While validated, the exposure definition of RA could result in misclassified cases. This misclassification concern was alleviated somewhat by design because patients had to have been prescribed an RA-related csDMARD to be included in the study. Unavailable covariates, such as disease severity, functional disability questionnaires (eg, the Disease Activity Score, 28, or the Health Assessment Questionnaire), and other unobserved covariates, may be associated with systemic differential influence across regions, patients, and prescribers. Individual-level data for patients and prescribers were used to minimize the risks of systemic bias and ecological fallacy.

## Conclusions

Prescription of the first biologic DMARD is a costly health care decision, with the choice to prescribe a biologic DMARD associated with large cost and clinical implications. Therefore, small changes in time to first biologic DMARD have major clinical and economic impacts. From a clinical perspective, the prescription of a biologic DMARD represents a transition to a more complex care plan, with less data to support the next prescription choice after the first biologic DMARD.

Although disparities in access to biologic DMARDs increased in this study, the overall prescription percentage decreased relative to the population of active medication users with RA. This suggests that despite rheumatologists prescribing fewer biologic DMARDs on average per patient per unit of time, variations in the prescription of biologic DMARDs continue to grow. The random-effects term in our models indicated a high amount of variance associated with the practice of rheumatologists within regions. These differences in prescriber preferences have unclear implications for patient outcomes but show that between-prescriber differences exist in health care delivery for patients with RA, despite identical health insurance coverage.

## 

## Notes

Supplement. **eAppendix.** Exposure and Outcome Medications **eFigure 1.** Study Accrual Diagram **eFigure 2.** Map of Ontario Local Health Integration Networks **eTable.** Random Effects for Physicians Within Regions
