# Supplementary material for: Association of Patient, Prescriber, and Region With the Initiation of First Prescription of Biologic Disease-Modifying Antirheumatic Drug Among Older Patients With Rheumatoid Arthritis and Identical Health Insurance Coverage
Source: JAMA Netw Open. 2019 Dec 6;2(12):e1917053. doi: 10.1001/jamanetworkopen.2019.17053 (PMC6902765; doi:10.1001/jamanetworkopen.2019.17053)
Supplement: Supplement. — eAppendix. Exposure and Outcome Medications eFigure 1. Study Accrual Diagram eFigure 2. Map of Ontario Local Health Integration Networks eTable. Random Effects for Physicians Within Regions [file jamanetwopen-2-e1917053-s001.pdf]

# Supplementary Online Content

Tatangelo M, Tomlinson G, Paterson JM, et al. Association of patient, prescriber, and region with the initiation of first prescription of biologic disease-modifying antirheumatic drug among older patients with rheumatoid arthritis and identical health insurance coverage. *JAMA Netw Open*. 2019;2(12):e1917053. doi:10.1001/jamanetworkopen.2019.17053

**eAppendix.** Exposure and Outcome Medications
**eFigure 1.** Study Accrual Diagram
**eFigure 2.** Map of Ontario Local Health Integration Networks
**eTable.** Random Effects for Physicians Within Regions

This supplementary material has been provided by the authors to give readers additional information about their work.

© 2019 Tatangelo M et al. *JAMA Network Open.*

**eAppendix.** Exposure and Outcome Medications

Exposure Medications

Methotrexate
Hydroxychloroquine
Leflunomide
Sulfasalazine

Outcome Medications

Etanercept
Rituximab
Tocilizumab
Infliximab
Golimumab
Anakinra
Adalimumab
Abatacept

Excluded Medications

Tofacitinib

© 2019 Tatangelo M et al. *JAMA Network Open.*

**eFigure 1.** Study Accrual Diagram

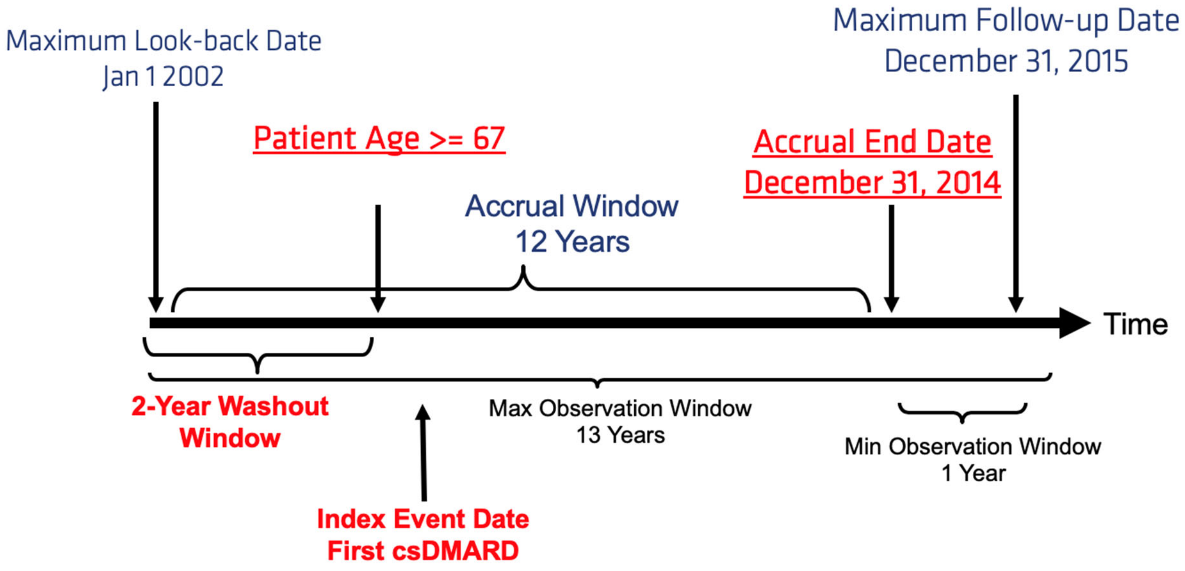


© 2019 Tatangelo M et al. *JAMA Network Open.*

**eFigure 2.** Map of Ontario Local Health Integration Networks

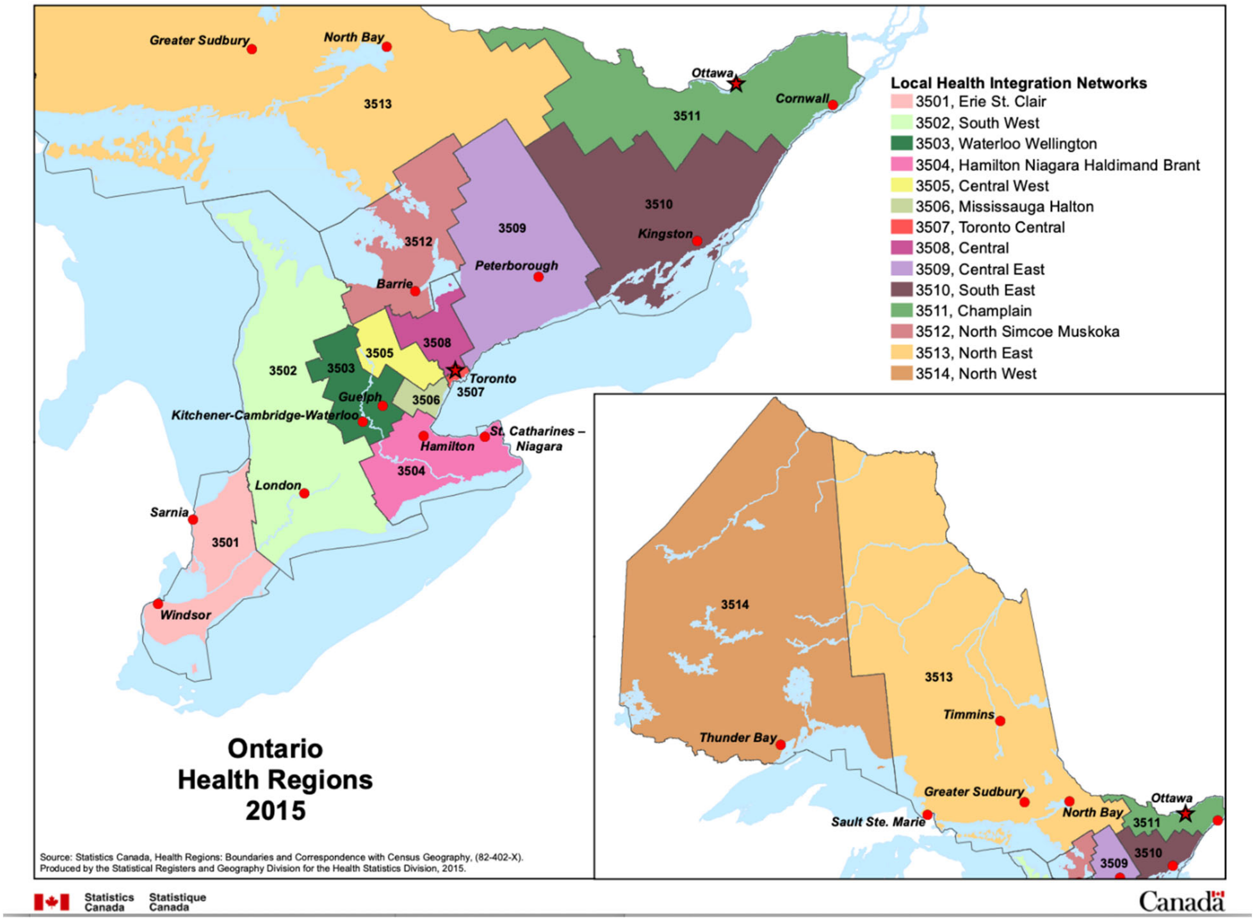


© 2019 Tatangelo M et al. *JAMA Network Open.*

**eTable.** Random Effects for Physicians Within Regions

Fully Adjusted Cox Model, Random Effects

| **Random Effects** | | | | |
|---|---|---|---|---|
| | **Standard Deviation** | **Variance** | **% Difference in Within Model Variance** | **% Difference in Between Model Variance** |
| **Basic Model (Age, Sex, Calendar year)** | | | | |
| **Physicians within Regions** | 0.86 | 0.74 | - | - |
| **Region** | 0.29 | 0.084 | 10.46% | - |
| **Full Adjustment (All Variables Described in Table 2)** | | | | |
| **Physicians within Regions** | 0.81 | 0.65 | - | 88.1% |
| **Region** | 0.22 | 0.046 | 7.39% | 62.3% |

© 2019 Tatangelo M et al. *JAMA Network Open.*
